# Optimal vaccine schedules to maintain measles elimination with a two-dose routine policy

**DOI:** 10.1017/S0950268816002296

**Published:** 2016-10-20

**Authors:** A. McKEE, K. SHEA, M. J. FERRARI

**Affiliations:** Department of Biology, Pennsylvania State University, PA, USA

**Keywords:** Maintenance of measles elimination, mathematical modelling, measles (rubeola), vaccination (immunization), vaccine policy development

## Abstract

Measles was eliminated in the Americas in 2002 by a combination of routine immunizations and supplementary immunization activities. Recent outbreaks underscore the importance of reconsidering vaccine policy in order to maintain elimination. We constructed an age-structured dynamical model for the distribution of immunity in a population with routine immunization and without disease, and analysed the steady state for an idealized age structure and for real age structures of countries in the Americas. We compared the level of immunity maintained by current policy in these countries to the level maintainable by an optimal policy. The optimal age target for the first routine dose of measles vaccine depends on the timing and coverage of both doses. Similarly, the optimal age target for the second dose of measles vaccine depends on the timing and coverage of the first dose. The age targets for the first and second doses of measles vaccine should be adjusted for the post-elimination era, by specifically accounting for current context, including realized coverage of both doses, and altered maternal immunity. Doing so can greatly improve the proportion immune within a population, and therefore the chances of maintaining measles elimination, without changing coverage.

## INTRODUCTION

Measles, a viral illness, infects millions of children every year and currently results in more than 100 000 deaths per year in children aged <5 years [1, 2]. As such, it is an important target for global eradication [3]. This eradication process includes two key components – achieving local elimination where the disease is present and maintaining elimination where the disease is absent. Different combinations of routine immunization strategies and supplemental immunization campaigns are used to achieve and maintain a high level of immunity [4]. Regional success was achieved when measles was eliminated from the Americas in 2002 using a combination of a two-dose routine immunization strategy with periodic supplemental immunization campaigns [5]. While endemic disease has not re-emerged, recent outbreaks, such as the outbreak in and around Disneyland, California from December 2014 to February 2015 [6], have cast doubt on the continued ability to maintain elimination. Optimizing the design of these vaccine strategies to maintain elimination in the Americas and achieve it worldwide is critical for continued success, for the eventual global eradication of measles, and for the end of childhood mortality attributable to measles.

Since elimination was achieved in 2002, maintenance of elimination in the Americas has involved two routine doses of vaccine administered to children who come to a clinic at specific target ages. These age targets for routine immunization have changed very little since measles was endemic in the Americas [7]. Conventionally, the timing of these doses is considered to be dependent on two underlying factors [8, 9]. The first is maternal immunity; infants born to immune mothers are born with measles IgG antibodies, which are passively transferred through the placenta before birth [10]. Infants are born with these antibodies regardless of whether their mother was vaccinated or naturally infected, although the initial titre is generally lower in children of vaccinated mothers [11]. While these maternal antibodies provide infants some protection from the disease, they interfere with the efficacy of the vaccine and infants vaccinated before antibody titre has dropped below a threshold level will not be effectively immunized [12, 13]. The second factor is the force of infection in the local population. Infants must be vaccinated before they become infected with, and potentially die from, measles [9]. Where measles incidence is high, children are likely to be exposed to infection earlier in life; thus it is more important to vaccinate children at younger ages. This second factor is absent in a disease-free setting, as is the case when measles elimination is being maintained.

Recent work suggests that the selection of these target ages may also depend on an additional context-dependent factor: demography [14]. If too many children fall below the age of first vaccination, there will be a large population of infant susceptibles contributing to the proportion of the overall population that is susceptible, thus decreasing the chances of maintaining measles elimination. Thus, even in the disease-free setting, this provides an upper bound on the age target for vaccination in order to maintain population level immunity above the herd immunity threshold.

Here, we show that the optimal age target may also depend on the coverage of the first and second doses. If coverage of the first dose is poor, the timing of the second dose should be adjusted to compensate, to account for the relatively large proportion of susceptible children between the first and second target ages of vaccination. If the coverage of the second dose is poor, the timing of the first dose should be adjusted to maximize efficacy, relative to the waning of maternal immunity, to compensate for the low probability of a second-dose opportunity.

Each of these context-dependent factors can create immunity gaps between apparent vaccine coverage and actual population immunity [14]. Unfortunate combinations of these factors can result in larger gaps than might otherwise be expected. For example, long duration of maternal immunity leads to low efficacy of the first dose at any given age, and if the timing of the second dose is not adjusted accordingly, a large population of individuals will remain susceptible between the first and second doses.

As a result of these immunity gaps, reported administrative coverage can greatly overestimate the true level of immunity within the population. In the absence of serological surveys, it is hard to know these true immunity levels in any population. When coverage is apparently high (not accounting for these context-dependent factors) and disease incidence appears low, it can be easy to assume that the population threshold for elimination is being maintained. However, the absence of disease is not the absence of risk. Many places have seen large unexpected outbreaks after years of apparently good coverage and low incidence, such as Brazil in 1997 [15], Burkina Faso in 2009 [16], Malawi in 2010 [17], Wales in 2012 [18], and Brazil in 2013 [19], among others. Such unexpected outbreaks are indicative of an unrecognized gap between coverage and population immunity.

At the country level, specific selection of age targets can account for these factors to reduce local susceptibility and therefore improve the chances that elimination will be effectively maintained. By explicitly accounting for age structure, country-specific variations in maternal immunity, and the expected coverage of each dose, age targets can be chosen that minimize the total proportion of individuals left susceptible. In this paper, we use a discrete-time age-structured population model for the distribution of immunity in a disease-free population with two routine doses, and analyse the equilibrium states of this model. We use this model to find the combination of age targets that minimizes the susceptible population given a specified combination of age structure, maternal immunity and coverages. We also use the model to explore the immunity gap between apparent coverage and actual population immunity, and how the size of this gap changes based on age structure, coverage of each dose and age targeting, although we generally ignore operational constraints. We use the results to suggest the source of some discrepancies between apparent coverage and disease risk. Further, we suggest that changing age targets may address these discrepancies, and thus help to maintain elimination in currently measles-free settings, such as the Americas.

## METHODS

We developed an age-structured model for immunity within a human population, using 131 age groups. Age groups are divided monthly up to 5 years of age, and then yearly up to 75 years. Individuals within these age groups are then further divided into one of three immune classes: maternally immune, susceptible, or successfully immunized. As we are concerned with maintaining measles elimination, we omit the disease process (there are no classes for individuals who are infectious or immune as a result of infection).

In this model, we track the immune status of individuals via these classes through life. Vaccines administered at any age have some rate of primary vaccine failure, as individuals may fail to seroconvert when receiving vaccination. One major cause of primary vaccine failure is maternal immunity. Individuals born to susceptible mothers are born to the first susceptible class, while individuals born to immune mothers are born to the maternally immune class, since they are born with antibodies that confer protection while their immune system develops [10], but also interfere with the efficacy of the vaccine. The maternal antibody titre wanes over time [11], so a smaller proportion of individuals in any older age group will retain this maternal immunity, and therefore these individuals will have a lower rate of primary vaccine failure. Vaccines are administered at some initial target age, usually before all individuals are susceptible, so only a proportion of the vaccines are effective (which we assume is equal to the proportion of that age group that was never or is no longer maternally immune). A second dose of vaccine is administered at a second target age, to individuals independently of whether they had the first dose. While the rate at which maternal immunity wanes is likely dependent on country, as it depends on the immune status of the average mother [11, 20], we assume that maternal immunity wanes exponentially with a mean at 3 months for the purpose of our model, and use this function as a proxy for the age-specific rate of primary vaccine failure. This function leads to vanishingly small rates of failure in older age groups (see Supplementary material for a sensitivity analysis of the rate at which maternal immunity wanes). We also assume a constant rate of primary vaccine failure of 5% across all age groups (which may arise from issues such as cold chain disruption), although we ignore all other operational constraints.

By tracking these immunizing processes throughout an individual's life, we can calculate the proportion of adults that have been successfully immunized, which will give us the proportion of infants in the next generation that will be born with maternal immunity. By solving for the steady state, we can find the stable proportion of infants born with maternal immunity in a disease-free setting.

The proportion of individuals who have been successfully immunized is the simply the sum of the proportion of individuals for whom the first dose was immunizing and the proportion of remaining susceptible individuals for whom the second dose was immunizing. That is, the proportion successfully vaccinated in generation *T, V*_*T*_, is:

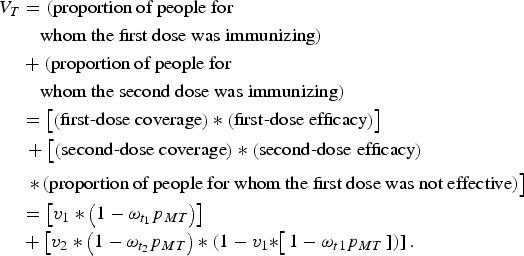


Here, first-dose coverage is *v*_1_, the second-dose coverage is *v*_2_; *ω*_*t*1_ and *ω*_*t*2_ are the proportion of individuals retaining maternal antibodies at the first and second age targets, respectively – we assume here that maternal immunity wanes exponentially with a mean at 3 months [21]. The proportion of individuals born with maternal immunity in generation *T* is *p*_*MT*_. Since vaccination is the only source of immunity, the proportion of individuals born with maternal immunity in generation *T* + 1 is simply *V*_*T*_. We can then solve for the equilibrium and get:

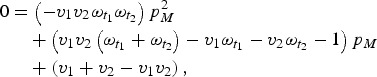

which we can then solve to find *p*_*M*_.

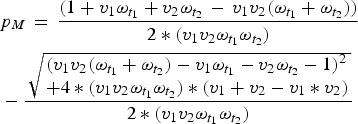


Once we know the equilibrium proportion of individuals born with maternal immunity, we can find the distribution of immunity throughout the age-structured population. We calculate the difference between this value and the expected coverage of the vaccine (*v*_1_ + *v*_2_ – *v*_1_*v_2_), to find the immunity gap caused by maternal immunity, local population age structure, and vaccine age targets. We can then choose optimal age targets for specific coverages by minimizing this immunity gap.

In this work, we first examine the effects of coverage of each dose on the optimal targets for an idealized developing country age structure, i.e. a concave age structure where a constant proportion of individuals die each year. We then perform the same optimization for a range of coverages on real age structures [22] representing countries in the Americas (specifically for all countries in North and South America for which age targets for two routine doses and age structure were readily available), chosen because these countries are in the process of maintaining measles elimination. We also compare the population immunity achieved by our optimization to that achieved by the real age targets on these real age structures [22, 23].

## RESULTS

**Fig. 1.**
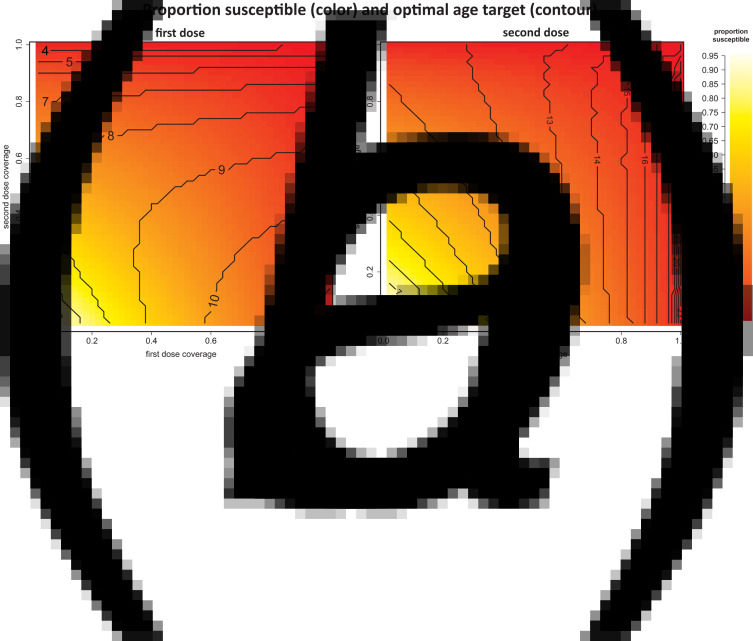
The optimal ages in months (shown by the contours), and maintained proportional susceptibility (shown by the colour scale) for a range of first- and second-dose coverages, varying independently, in a population with idealized developing age structure. (*a*) The optimal age for the first dose. Notably, the optimal age of the first dose depends heavily on the coverage of the second dose. (*b*) The optimal age for the second dose. Notably, the optimal age of the second dose depends heavily on the coverage of the first dose.

The coverage of each dose has a significant effect on the optimal target ages for the first and second doses and the resulting proportion of the population that remains susceptible with a generic developing country age structure (Fig. 1). Susceptibility varies straightforwardly with coverage; as coverage of either dose increases, the remaining proportion susceptible decreases. In the lower left of both panels, the coverage of both doses is low, and population immunity is similarly low. In the upper right of both panels, the coverage of both doses is high, and population immunity is high. In the lower right, where first-dose coverage is high and second-dose coverage is low, and the upper left, where first-dose coverage is low and second-dose coverage is high, population immunity is similarly high. The optimal target ages, shown by the contours, also vary with coverage of both doses. The optimal target age for the second dose varies more with first-dose coverage (Fig. 1*b*) than second-dose coverage; i.e. the contours in Figure 1*b* run nearly parallel to the second-dose coverage axis but indicate a steep change in optimal second-dose timing for a relatively small change in first-dose coverage. The optimal target age for the first dose is strongly dependent on first-dose coverage when second-dose coverage is low, but depends more strongly on second-dose coverage when second-dose coverage is high (Fig. 1*a*).

**Fig. 2.**
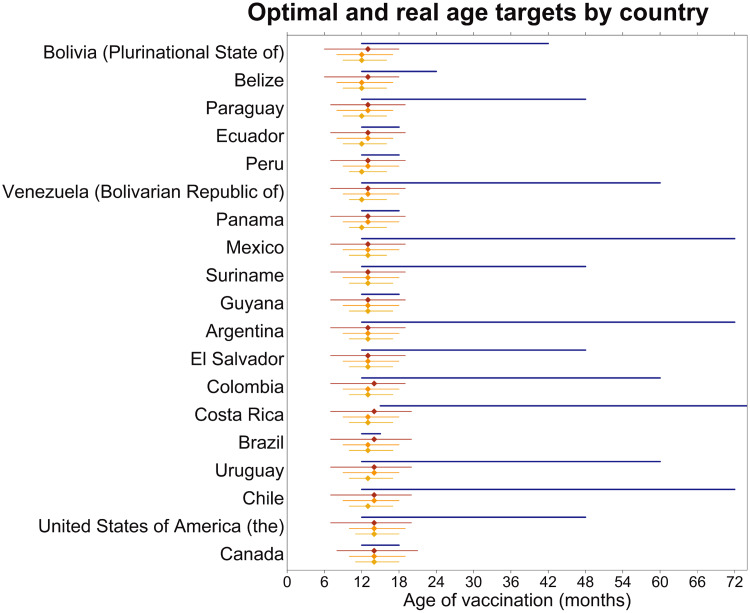
The real target ages (the blue line), the optimal target ages with 100% coverage of two doses (the red line), the optimal target ages with 90% coverage of two doses (the orange line), and the optimal target ages with 80% coverage of two doses (the yellow line). The endpoints of each line represent the first- and second-dose age targets, respectively, for each country and policy. The optimal target ages for a single-dose vaccine schedule with each of these coverages are shown by the diamond on each line. In all cases, the difference in age target between the first and second doses is smaller with lower coverages. In all cases, the optimal first age of vaccination is younger than the current recommendation, and in most, the optimal second age is also younger than the current recommendation. The optimal single-dose ages correspond well with the current recommendation for the first dose. The countries have been ordered by proportion of the population made up by children aged <5 years, with Bolivia having the most children aged <5 years and Canada having the fewest.

We test these ideas with real age structures, using age structures from countries in the Americas. For every country, we find the optimal age for the first and second doses for a range of coverages (80%, 90%, 100%) for both doses, assuming each dose has equal coverage (Fig. 2, Supplementary Table S1). For all countries, the higher the coverage, the longer the recommended time between doses. The model-recommended age for the first dose was at a younger age than current policy in all countries, and the model-recommended age of the second dose was also at a younger age than the current policy in most countries; Brazil, Canada and Peru are exceptions that recommend second-dose administration before age 2 years. We also find the optimal single-dose age target – i.e. the one-dose strategy that minimizes the proportion susceptible – for this range of coverages in all these countries (Fig. 2). Interestingly, this is usually close to current policy recommendations for the first dose – around 12 months. In the Supplementary material we present a comparison of these idealized coverage levels with current age targets and coverage of the first and second dose of measles containing vaccine (MCV1 and 2, respectively).

**Fig. 3.**
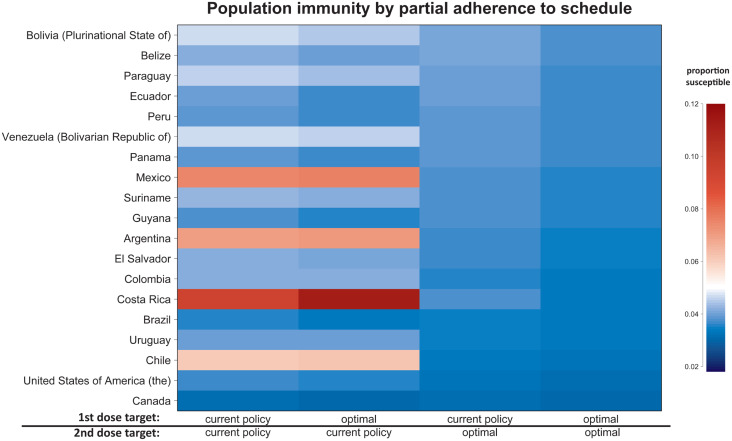
The population immunity by partial adherence to schedule for countries in the Americas with two recommended age targets of vaccination. Red indicates a population immunity below 95%, the commonly accepted threshold for maintaining elimination, and blue indicates a population immunity above 95%. In the case where only one dose is optimal, the other dose is administered at the currently recommended age target.

We also calculate the differences that changing age targets make in population immunity (Fig. 3). All countries are expected to see a reduction in the proportion susceptible using our model-specified optimal age targets for both the first and second doses in place of current age targets. However, implementing our optimal age target for only one dose, but not the other, can be detrimental in some cases. For example, in Costa Rica, our model predicts that the current policy of vaccinating at 15 months and 7 years would maintain population immunity at 90·6%, given 90% coverage. If only the first-dose age target in Costa Rica were changed to our model-recommended optimum of 9 months, the level of population immunity maintained would be reduced to 88·9%. If the second-dose age target in Costa Rica were reduced to our model-recommended optimum of 19 months, with the first-dose age target held at the current recommendation, population immunity would be improved over that maintained by current policy to 96·3%. Finally, if the age targets of both doses in Costa Rica were changed to our model-recommended optima, population immunity could be maintained at 96·7%. While this is an illustrative example where changing the age target of the second dose can markedly improve population immunity, note that these policy recommendations should not be implemented without further country-specific analysis, as our model ignores several operational constraints.

In general, if the second dose is recommended relatively late in life, say at age 6 years as it is in Argentina, lowering the recommended age for the first dose from 12 months to 8 months would reduce first-dose efficacy and expand the duration of susceptibility between doses, thereby reducing population immunity. Conversely, if both doses are already administered relatively close together, as they are in Peru where they are recommended at 12 and 18 months, lowering the second-dose age target to 16 or 17 months without adjusting the first-dose age target can reduce second-dose efficacy without substantially reducing the duration of the susceptibility window between doses. However, in most countries, reducing the age target of the second dose alone will result in an increase in the proportion of the population that is immune. Changing age targets may be enough to make measles elimination maintainable in places where it was not, without changing coverage, most notably in Argentina, Chile, Costa Rica, Ecuador and Mexico, although the maximum maintainable population immunity is still dependent on age structure.

## DISCUSSION

Measles is a highly lethal disease, killing hundreds of children worldwide each day. With more than 1000 cases in the Americas in the first half of 2015 [24] and hundreds of cases spanning five outbreaks in the United States alone [25], re-emergence is a serious threat. It is important that we focus our attention on optimizing current policy in order to prevent continued re-emergence, and maintain elimination. By reconsidering vaccination policy in the context of the continued absence of both disease and supplemental immunization activities (SIAs), we can increase the proportion immune within the population and better maintain elimination.

Here we show that the optimal target age for each dose depends on the coverage of the other; thus optimal scheduling should not consider the doses independently. Optimal selection of both age targets together may have a large impact on the resulting population immunity. Optimal coverage for both doses is 100%, and vaccination efforts should, and do, aim to maximize coverage. However, realized coverage is often lower than administrative goals. If first-dose coverage is discovered, by coverage surveys or other mechanisms, to be low due to poor compliance with, or effectiveness of, vaccination programmes, the age target of the second dose should be adjusted accordingly, and vice versa. These adjustments to the timing of doses may markedly improve population immunity without changing coverage at all. Consequently, the target ages of vaccination should be adjusted according to estimated levels of programme efficacy, vaccine abstention and non-compliance with vaccine policy, in order to maximize the population immunity achieved with current coverage.

When applied to real age structures from the Americas, our model optimization gives recommendations that differ from current strategies in most countries. In nearly all cases, our model recommends lowering the age target for both doses. The optima for a two-dose strategy look very different from current policy, although they match the single-dose optimum, which happens to be similar to the current recommended first-dose age target, when coverage for the second dose is set to zero. However, the similarity between these targets is coincidental as current first-dose targeting was chosen to balance maternal immunity and force of infection in the context of endemic disease and SIAs [8], while our model optima were chosen to balance maternal immunity and age structure. Adjusting current policy to account for the current epidemiological and management context, even partially, may have a significant impact on the feasibility of maintaining measles elimination in these countries.

In most countries, simply decreasing the age target of the second dose may markedly improve population immunity by minimizing the susceptible population between doses. The exception to this is if current policy already recommends the second dose relatively early, as it does in Bolivia, Belize and Peru. In these countries, significant reductions in the remaining susceptible proportion of the population can be had by reducing the age target of the first dose, but reducing the second-dose age target without adjusting the first-dose age target reduces the efficacy of the second dose with little benefit. The minimum susceptible proportion under any management strategy in these countries still depends on the proportion of children aged <5 years. Note that Canada does not face the same issue as Boliva, Belize and Peru, despite also having a relatively early second-dose recommendation, because of its age structure. When a large fraction of the population falls below and between the age targets for vaccination, a low level of susceptibility can be hard to maintain, but this can be mitigated by selecting locally optimal, country-specific age targets.

Interactions between age structure, maternal immunity and age targets for vaccination can cause gaps between apparent coverage and the resulting population immunity [14]. These gaps may provide alternate explanations for cases where measles control has failed in the Americas, such as in São Paulo during the 1997 outbreak [15]. Rather than simply looking for failures in vaccine coverage, such as issues with vaccine scheduling and current vaccine delivery mechanisms, it may be important to reconsider the target ages for vaccination as well. Improvements in population immunity are possible by adjusting scheduling to account for partial compliance, especially in countries where compliance with vaccine policy has been fairly consistent over time and is unlikely to change as a result of changes in scheduling.

There are a number of operational caveats not explicitly considered in our model. Our results are sensitive to, and conditional on, a given function for maternal immunity. The real rate at which maternal immunity wanes in a specific country, and therefore the age-specific rate of primary vaccine failure, should be determined and explicitly considered as part of a re-evaluation of current policy. The optimal age target should be estimated based on the anticipated age-specific response to vaccination, as well as population level measures, such as coverage and age structure. This age-specific response will vary from country to country, as time since elimination (and therefore the ratio of vaccinated to naturally immune mothers) varies from country to country. It can be difficult to determine the age-specific waning of maternal immunity, as it would require high resolution longitudinal serosurveys in children not exposed to disease or vaccination. Similarly, directly measuring the age-specific response to vaccination would require detailed cohort studies. If maternal immunity wanes slowly, so that older children have a relatively high rate of primary vaccine failure, age targets should be kept the same or increased. However, if maternal immunity wanes more quickly, as it is likely to do due to the relatively high proportion of mothers who are vaccinated rather than naturally immune, then the age targets should be shifted earlier (Fig. 2).

The results presented here reflect a mathematical optimum and do not explicitly account for the logistical constraints of vaccine programme implementation. In our model, we assumed coverage was independent of age target selection, but in reality, changing age targets will likely change coverage, for a variety of reasons [26]. For example, if a change in age target requires an additional clinic visit from parents, then many parents may fail to comply. Similarly, multivalent vaccines (the measles vaccine is typically packaged with mumps and rubella vaccines) may impose constraints; that is, changing the age target for the measles vaccine could require either decoupling it from the mumps and rubella vaccines or changing the ages at which those partner vaccines are administered. This might impose a large disruption on vaccine schedules, and could require children to receive an additional shot, with attendant additional complications in supply chains. Nevertheless, our work presents a theoretical optimum and a framework to evaluate an optimal age target given known maternal immunity and operational constraints on possible age targets.

We also ignore SIAs in this model. SIAs are periodic campaigns where everyone within a target age range is vaccinated. Some countries in the Americas still perform these campaigns [27]. Data on the details and post-campaign assessments of coverage among the unvaccinated population are sparse, and performing our optimization to account for infrequent campaigns of variable coverage would provide less generalizable results. SIAs provide an additional source of immunity and thus could also affect optimal age targets, which should be considered before implementing any change in policy if SIAs are anticipated to happen frequently or at regular intervals. Additionally, we note that SIAs could help to smooth transient disruptions in immunity caused by changing age targets.

These results are the product of an equilibrium analysis in the absence of disease. Disease absence is important to consider when planning for the maintenance of elimination, as outbreaks provide an additional immunizing factor and can help maintain high levels of population immunity – considering the situation in the absence of disease provides us with a conservative analysis of the levels of immunity within a population. A more realistic model could include disease and demographic dynamics, including seasonality of the disease, which our model excludes, in order to capture the historical dynamic changes in population immunity following measles elimination, but would likely provide more optimistic results than our model. We would strongly recommend a more detailed analysis on a country by country basis, using locally appropriate assumptions about demographic structure, historical coverage levels and waning of maternal immunity, before policy is changed. Nevertheless, our results support the potential benefit of such a re-analysis, especially given the absence of endemic disease and SIAs, and provide a conservative estimate of the levels of immunity maintainable in a population. After more than a decade of absence, and using data on actual vaccine uptake, future policy should consider anticipated coverage of both doses in order to avoid re-establishment of measles and to prevent future mortality.

## Supplementary material

For supplementary material accompanying this paper visit http://doi.org/10.1017/S0950268816002296. click here to view supplementary material
